# O cuidado ao surdo no serviço de saúde: um clamor silenciado

**DOI:** 10.1590/0102-311XPT045224

**Published:** 2025-01-27

**Authors:** Luana Paula de Figueiredo Correia, Márcia de Assunção Ferreira

**Affiliations:** 1 Universidade Federal do Rio de Janeiro, Rio de Janeiro, Brasil.

**Keywords:** Psicologia Social, Pessoas com Deficiência Auditiva, Surdez, Língua de Sinais, Assistência à Saúde, Social Psychology, Persons with Hearing Impairments, Deafness, Sign Language, Delivery of Health Care, Psicología Social, Personas con Deficiencia Auditiva, Sordera, Lengua de Signos, Atención a la Salud

## Abstract

O objetivo deste estudo é analisar as representações sociais do cuidado à saúde de pessoas surdas por profissionais de saúde. Para tal, desenvolveu-se uma pesquisa de caráter qualitativo, com aplicação da Teoria das Representações Sociais em sua abordagem processual. Foram realizadas entrevistas semiestruturadas em profundidade com profissionais enfermeiros, técnicos de enfermagem, fisioterapeutas e médicos de uma unidade de saúde hospitalar no Município de Porto Velho, Rondônia, Brasil. Os dados produzidos foram submetidos à análise lexical por meio do software Alceste. Os resultados indicam que as representações da assistência a pessoas surdas na saúde possuem três dimensões: simbólica, associada à interdição da comunicação; afetiva, demonstrada por meio de sofrimento, angústia e receio em atender usuários surdos; e atitudinal, em que os profissionais se utilizavam de estratégias com vistas ao estabelecimento de comunicação para realização da assistência.

## Abstract (en)

This study analyzes the social representations of healthcare for deaf individuals by healthcare professionals. To this end, a qualitative study was conducted, applying the Theory of Social Representations in its procedural approach. In-depth semistructured interviews were conducted with nurses, nursing technicians, physical therapists, and physicians from a health facility in Porto Velho, Rondônia State, Brazil. The data produced were subjected to lexical analysis using the Alceste software. The results indicate that the representations of care for deaf individuals in healthcare take place through three dimensions: the symbolic dimension, associated with the interdiction of communication; the affective dimension, manifested via suffering, anguish, and fear in assisting deaf patients; and the attitudinal dimension, in which professionals employed strategies aimed at establishing communication to provide care.

## Abstract (es)

El objetivo de este estudio es analizar las representaciones sociales del cuidado a la salud de las personas sordas por parte de los profesionales de la salud. Para ello, se desarrolló una investigación de carácter cualitativo, aplicando la Teoría de las Representaciones Sociales en su enfoque procedimental. Se realizaron entrevistas semiestructuradas en profundidad con profesionales de enfermería, técnicos de enfermería, fisioterapeutas y médicos de una unidad de salud hospitalaria de la ciudad de Porto Velho, Rondônia, Brasil. Los datos producidos se sometieron a un análisis léxico utilizando el software Alceste. Los resultados indican que las representaciones de la asistencia a las personas sordas en la salud tienen tres dimensiones: simbólica, asociada a la prohibición de la comunicación; afectiva, manifestada mediante el sufrimiento, la angustia y el miedo a atender a usuarios sordos; y actitudinal, en la que los profesionales utilizaban estrategias para establecer comunicación para brindar asistencia.

## Introdução

Mundialmente, mais de 430 milhões de pessoas vivem com perda auditiva severa a profunda e até 2050 esse número pode crescer para quase 700 milhões ^1^. No Brasil, cerca de 1,1% da população, ou seja aproximadamente 10 milhões de pessoas, possuem deficiência auditiva. Destas, 21% de grau severo a profundo, o que, em geral, acarreta em comprometimento de atividades cotidianas e limitações, especialmente linguísticas ^2^, devido à carência de acessibilidade por meios comunicativos visuais e, no caso de surdos usuários de língua de sinais, acessibilidade linguística.

Evidencia-se a questão dos surdos como parte da Diversidade Surda, a qual é conhecida por meio de pessoas surdas usuárias de língua de sinais (sinalizantes), implantadas (usuárias de implante coclear), oralizadas (uso da língua portuguesa para comunicar-se) e usuárias de aparelho auditivo, sendo reconhecidos como minoria sociocultural e linguística. Ao legitimar a heterogeneidade desse grupo, ressalta-se que esta pesquisa centra como público de interesse os surdos sinalizantes ^3^.

Entre as políticas públicas que tratam da língua de sinais e inclusão de pessoas surdas no Brasil, tem-se o *Decreto nº 5.626/2005* ^4^, que regulamenta a Lei de Libras, de 2002. Essa lei tem mais de 15 anos e traz um campo só para a acessibilidade na saúde, mas ainda se observa um distanciamento entre o que está publicado e sua aplicação. Há também uma contradição nesse Decreto: ao mesmo tempo que exige serviços de saúde com profissionais capacitados em língua de sinais para o atendimento à pessoa surda, exige como obrigatória a disciplina de Libras (Língua Brasileira de Sinais) somente para a área de Fonoaudiologia entre os cursos de graduação em saúde.

Outro impasse pode ser observado quando, por um lado, houve crescimento da oferta dos serviços de concessão de aparelhos auditivos e terapia fonoaudiológica para habilitação/reabilitação auditiva como consequência de políticas públicas, como o Plano Viver sem Limite e a criação da Rede de Cuidados a Pessoa com Deficiência, porém, por outro, a acessibilidade comunicacional e o cuidado inclusivo pouco evoluíram. O foco em intervenções reabilitadoras suplantando ações com vistas à integralidade do cuidado revela a força do modelo organicista da deficiência, que dialoga intimamente com a formação biomédica na saúde.

Na saúde, a dificuldade para estabelecer um meio de comunicação comum atinge não só os usuários do serviço, mas também os profissionais, provocando angústia e ansiedade ^5^. Além disso, a compreensão sobre a pessoa surda usuária de língua de sinais pode se basear em estigmas, próprios de senso comum, que interferem no cuidado ^6^.

Cuidar de uma pessoa surda usuária de língua de sinais em um serviço de saúde suscita reflexões e debates, porque a comunicação oral é imperiosa na nossa sociedade e a língua de sinais não é de dominada por profissionais, na maioria das vezes. As interdições na comunicação reconfiguram o atendimento e refletem, dialeticamente, em novos atos de cuidar, permeados por fenômenos afetivos, comportamentais e culturais. Nesse sentido, o cuidado à saúde da pessoa surda usuária de língua de sinais se configura em um fenômeno passível de abordagem pela via das representações sociais.

As representações sociais se expressam nos valores que orientam as relações entre as pessoas e o mundo, influenciando condutas, comportamentos e comunicações sociais ^7^; logo, orientam práticas e justificam ações, além de facilitar a comunicação entre os grupos ^8^.

A questão a ser investigada é: Quais são as representações sociais dos profissionais de saúde sobre o cuidado ao surdo usuário de língua de sinais e suas repercussões no cuidado à saúde desse grupo humano? Esta justifica-se pela necessidade de investigar tais representações e suas influências no cuidado à pessoa surda usuária de língua de sinais, com intuito de contribuir com a qualificação da assistência e formação dos profissionais. O objetivo deste estudo é analisar as representações sociais dos profissionais de saúde sobre o cuidado ao surdo usuário de língua de sinais e as potenciais repercussões no cuidado à saúde desse grupo humano.

## Método

Pesquisa qualitativa, do tipo analítico, com aplicação da abordagem processual da Teoria das Representações Sociais, teoria essa que tem sido empregada em diversos campos do conhecimento, destacando-se na saúde por abordar fenômenos socioculturais, relacionados à vida social de grupos humanos.

O campo correspondeu a um hospital estadual situado em Rondônia, Brasil, e foram convidados para participar os membros da equipe de saúde das categorias médica, enfermagem, psicologia e fisioterapia. Os critérios de inclusão foram: profissionais ativos, com no mínimo um ano de experiência clínica. Os de exclusão foram: afastados do serviço por férias ou licença de qualquer natureza durante o período de produção de dados. Não houve participação de profissionais psicólogos, pois nenhum dos convidados aceitou participar da pesquisa.

Para captar os potenciais participantes, fez-se um levantamento dos profissionais no setor de recursos humanos da instituição, quais profissões apresentavam maior possibilidade de contato com usuários surdos, suas áreas e postos de atuação e turnos de trabalho, seguido de visitas aos postos com uma breve apresentação da pesquisadora. Apesar de não ser critério de inclusão, fez-se uma sondagem junto aos profissionais sobre a experiência de ter atendido pessoas surdas no seu exercício profissional em qualquer instituição em que trabalha ou já tenha trabalhado ou realizado curso de Libras, para que se abrangesse profissionais com e sem experiência no atendimento desses usuários, sem a intenção de estabelecer comparações entre grupos. Com essa sondagem, foram identificados que aqueles que atenderam pessoas surdas, o fizeram no campo da pesquisa e que os setores de maior contato com pessoas surdas eram a maternidade e a clínica médica.

Pela dificuldade de recrutar profissionais com experiência no atendimento a pessoas surdas, aplicou-se a estratégia da “bola de neve” ^9^ a partir de uma enfermeira da maternidade (semente), que indicou mais uma enfermeira e três técnicas de enfermagem. Outros profissionais foram indicados por essas primeiras participantes. A produção de dados ocorreu no período de julho a outubro de 2019 e se encerrou com uma amostra de 37 pessoas, após aplicação do critério de saturação dos dados, com análise preliminar em face do objetivo da pesquisa ^10^.

Em entrevista individual, aplicou-se um instrumento com questões fechadas sobre perfil pessoal, de formação e atuação profissional, para melhor compreensão do contexto dos participantes, bem como com questões abertas e semiestruturadas sobre os seguintes temas: “conhecimento sobre a pessoa surda e a língua de sinais”, “assistência à saúde da pessoa surda” e “formação para o cuidado à saúde da pessoa surda (cursos livres de Libras, pós-graduação em Libras, disciplina de Libras na graduação)”, tendo duração média de 35 minutos.

O processamento dos dados provenientes das questões abertas foi feito pelo software de análise lexical Alceste 2012 (http://www.alcestesoftware.com.br/), que aplica recursos de estatística cruzados com uma teoria da linguagem na identificação de palavras que evidenciem mundos lexicais que informam as principais ideias e sentidos sobre o objeto investigado, evidenciando as palavras mais significativas dos discursos, baseado na coocorrência lexical ^11^.

O texto das entrevistas foi processado em *corpus* único, composto por 37 entrevistas, denominadas de unidades de contexto inicial (UCI). Após o processamento, houve 86% de aproveitamento do *corpus*.

Para análise, utilizou-se os dendrogramas gerados com a classificação hierárquica descendente (CHD) e ascendente (CHA), a rede de relação entre as palavras a partir do léxico mais representativo da CHD, subsidiados pelos textos das unidades de contexto elementares (UCE), que correspondem aos excertos selecionados dos discursos. Aos dados das questões fechadas aplicou-se análise estatística simples e percentual.

A identidade dos participantes foi preservada por códigos alfanuméricos, a saber: M - médico, E - enfermeiro, TE - técnico de enfermagem, F - fisioterapeuta, seguido do número sequencial da entrevista; além de características como atendimento ou não de pessoas surdas (APS e NAPS), formação ou não em Libras (FL e NFL), convive ou não com pessoas surdas (CPS ou NCPS).

Atendeu-se a *Resolução nº 466/2012* do Conselho Nacional de Saúde, com aprovação pelo Comitê de Ética em Pesquisa da Escola de Enfermagem Anna Nery e Hospital Escola São Francisco de Assis da Universidade Federal do Rio de Janeiro (protocolo nº 2.763.298). Todos os participantes assinaram o Termo de Consentimento Livre e Esclarecido.

## Resultados

Dos 37 participantes, 89% eram mulheres; 27% fisioterapeutas, 27% enfermeiras, 24% médicos e 22% técnicos de enfermagem; 76% realizaram atendimento à pessoa surda; 51% tinham alguma formação em Libras, destes 52% havia cursado disciplina de Libras na graduação, 26% curso livre e 21% havia realizado disciplina e curso livre; 42% dos participantes convivem com surdos, principalmente em âmbito profissional (escola bilíngue para surdos de Porto Velho como campo de estágio e em clínica particular), 33% no âmbito familiar e 25% no religioso (religião evangélica).

O *corpus* submetido à análise constituiu-se de 37 UCIs, o qual se subdividiu em 1.995 UCEs, formadas por 5.345 palavras distintas. Logo após, o software reduziu essas palavras em raízes distintas, compondo 1.995 UCEs, sendo selecionadas 1.664 para análise, distribuídas em três classes lexicais.

O cuidado a pessoas surdas, sua prática, crenças e sentimentos emergiu no conteúdo que formou a classe lexical 1. Essa classe correspondeu a 65% do *corpus* e foi formada por 1.064 UCEs e 73 palavras analisáveis típicas dessa classe. A palavra de maior associação estatística à classe foi *entend+*, como mostra o Quadro 1.

**Quadro 1:** Palavras representativas da classe 1 (1.064 unidades de contexto elementares), conforme o dendrograma da classificação hierárquica descendente (CHD), e respectivas associações estatísticas à classe. Rio de Janeiro, Brasil, 2019.

FORMA REDUZIDA	FORMA COMPLETA	**VALOR DE *PHI***
*entend*	entender, entende, entendam e entendem	0,18
*consegu*	consegue, conseguia, conseguem, conseguimos, conseguiam	0,17
*tent*	tentar, tentando, tenta, tentam, tentamos, tentado, tentasse	0,16
*comunic*	comunicação, comunica, comunicam, comunicado	0,15
*fic*	fica, ficado, ficam, ficamos, ficando, ficar, ficaram, ficasse	0,15
*fal*	falando, fala, falam, falado, falante, falantes, falada	0,14
*acompanh*	acompanha, acompanhada, acompanhado, acompanhando	0,13
*mae*	mãe(s)	0,12
*express*	expressa, expressado, expressam, expressando, expressão	0,12
*sab*	sabe, sabem, sabemos, saber, sabia, sabiam	0,11
*explic*	explica, explicação, explicada, explicado, explicamos	0,11
*difícil*	difícil	0,11
*olh*	olhada, olham, olhando, olhar, olhava, olhe, olho, olhou	0,10
*convers*	conversa, conversação, conversado, conversamos, conversando	0,10
*bebe*	bebê(s), bebezinho	0,10
*dor*	dor(es)	0,09
*ouv*	ouve, ouvia, ouvinte, ouvintes, ouvir, ouvisse, ouviu	0,09
*escut*	escuta, escutam, escutando, escutar, escutava, escuto	0,09
*normal*	normal, normalmente	0,09
*mesma*	mesma(s)	0,09
*escrev*	escreva, escreve, escrever, escrevesse, escreveu, escrevia	0,09
*deficiente_auditiv*	deficiente_auditiva(o)	0,09
*quer*	quer, querem, queremos, querer, queria, queriam, quero	0,08
*diferenc*	diferença(s)	0,08
*dificuldade*	dificuldade, dificuldades	0,08
*cheg*	chega, chegam, chegando, chegar, chegaram, chegasse	0,08
*filha*	filha	0,08

Fonte: dados da pesquisa.

O radical “*entend*”, cuja forma completa de maior frequência correspondeu a palavra “entender”, articula-se com os radicais e respectivas formas completas de maior associação estatística, em relação de proximidade nos discursos. Os termos “*consegu*”, “*fal*”, “*tent*” e “*comunic*” mostram as tentativas das pessoas surdas se fazerem entender por meio de suas falas ao tentarem se comunicar com os profissionais de saúde.

Os outros blocos de radicais com maior associação estatística à classe e suas relações com as formas completas mostram as dificuldades para se expressarem e a necessidade de ter acompanhante para que possam se explicar e receber as explicações dadas nos atendimentos de saúde. Os radicais de menor associação estatística exemplificam situações específicas vivenciadas pelas pessoas surdas e as estratégias aplicadas para a comunicação.

Identifica-se, portanto, que a classe 1 volta-se para a prática do cuidado ao surdo usuário de língua de sinais, e o quanto o valor dessa prática se debruça no ato comunicativo, mais precisamente no impedimento deste pela ausência de uma língua em comum para mediar a relação, o que demanda a presença de acompanhantes na consulta, ideia expressa na relação com os outros radicais, como o “*acompanh*”.

Os pensamentos expressos nos discursos, traduzidos nas UCEs, mostram questões referentes ao conhecimento, sentimentos e afetos, comportamentos, atitudes, ações e estratégias para promover o cuidado, que correspondem às dimensões das representações sociais: simbólica, afetiva e atitudinal.

“*A gente chama as pessoas pela doença, isso deixa a gente mais afastado delas. Eu acho que fica mais difícil tratar assim. Eu acho relevante, porque agora eles ganharam um pouco mais de notoriedade. A gente não via isso antigamente. Na verdade, a gente nem entendia que era surdo, entendia que era mudo, porque eles não falavam*” (F36, NFL, NCPS, APS).

“*Acho que o atendimento é igual, o fato de ser surdo não vai mudar todo o resto do organismo. A única diferença é o jeito que eu vou transmitir as informações para ela, e que ela vai transmitir as informações dela para mim*” (M8, NFL, NCPS, NAPS).

“[Referindo-se ao atendimento de haitianos] *‘Venham e aprendam o português, eles que estão na terra estrangeira’. Isso para mim foi um choque. Eu associo muito isso ao surdo. Quando alguém me fala: ‘não tenho paciência para falar com ela porque ela tem essa deficiência auditiva’, eu digo: ‘tudo bem, eu vou’, porque eu sei que se ela for, vai acontecer aquela cena que é muito desagradável, desconfortável para a gente*” (E1, NFL, NCPS, NAPS).

“[Referindo-se ao parto] *Eles levantam e mostram para a mãe e ela vê que o bebê nasceu e que está fazendo a expressão do choro, isso para ela poder participar daquele momento*” (M30, FL, CPS, APS).

“*Acho que houve inclusão porque elas foram atendidas. Teve assistência de enfermagem e médica, o que colhemos de informação, tentamos suprir todas as necessidades. Se houve falha, foi pela dificuldade da comunicação, mas o que foi colhido e o que a gente conseguiu conversar e se expressar incluiu a pessoa sim*” (E15, FL, NCPS, APS).

“*Acolher a gente acolhe, mas como ela merecia eu acredito que não pela tristeza no olhar, pelo medo que ela expressava. O corpo fala. Você olha nos olhos de alguém você sabe quando está assustado, desesperado. Na medida do possível foi tentado, mas ficou deficiente*” (TE43, FL, CPS, APS).

“*Não deixou de atender. É muito difícil saber se a pessoa surda foi acolhida. O acolhimento vai muito além de você saber compreender a pessoa. Eu sou ouvinte, você é surda, ela não consegue ouvir, eu não falo libras, mas eu tento entender, eu te trato com respeito, eu te trato com educação*” (E13, FL, CPS, APS).

“*Você fica impotente e restrito, mas eu acho que não conseguiria suprir todas as necessidades deles, porque se você não se comunica, é difícil. Às vezes, pela expressão facial, consegue detectar uma dor, ou um incômodo, mas saber de fato o que as pessoas sentem é só se comunicando, conversando, falando*” (E7, NFL, NCPS, NAPS).

“*Eu consegui algumas coisas. No outro dia chegou um familiar, conseguiu me auxiliar e eu consegui terminar o exame, mas, de início, consegui somente os principais pontos com a ajuda da internet*” (M14, FL, NCPS, APS).

## Discussão

As representações sociais do cuidado à saúde de pessoas surdas sinalizantes se expressam na falta de entendimento comunicacional. Para atuar na clínica, é indispensável acessar informações dadas pelos usuários, sejam objetivas ou subjetivas. A dificuldade comunicacional interdita o atendimento, compromete o processo de trabalho e reflete no cuidado, ao afastar tanto os profissionais dos surdos, quanto os surdos dos profissionais e do serviço de saúde.

A comunicação é determinante da qualidade e segurança da assistência e para garantir sua efetividade, os profissionais devem estar preparados e capacitados para instituir relações ordenadas, com vistas a propiciar a redução dos riscos. Utilizar-se de um conjunto adequado de informações evita danos ao usuário ^12^.

O processo de comunicação é complexo, dinâmico e flexível. Apresenta elementos estruturados que influenciam de forma negativa ou positiva, cujo propósito alinha-se ao entendimento entre as pessoas. Para esse fim, os envolvidos precisam estar dispostos e atentos ao ato comunicativo em sua totalidade, seja pelo que é verbalizado por fala ou escrita, assim como pelo que é observado e percebido ^13^.

A comunicação é fundamental no processo de cuidar, tem valor humanizador ao possibilitar o estabelecimento das relações interpessoais entre cuidador e pessoa cuidada, pois permite a escuta, o acolhimento e a instituição de vínculo. A ausência ou deficiência na comunicação interdita o cuidado.

A representação da igualdade nos atendimentos está centrada no argumento de que a patologia que acomete as pessoas surdas é a mesma das pessoas não surdas e, portanto, o tratamento será o mesmo. Porém, essa representação reduz o cuidado ao aspecto biológico, desconsiderando que a saúde resulta também de uma produção social, de inserção em territórios, expressa a história e a cultura em que se está imerso.

Essa redução mostra a força do paradigma biomédico, que resiste nos serviços, com uma visão de ser humano fragmentado, tendência a desprezar a multidimensionalidade e multiculturalidade humana, com enfoque na doença e cura, que nos remete à metáfora do homem-máquina, o que não atende ao princípio de integralidade em saúde ^14^.

A distinção na comunicação é condição suficiente para diferenciar o atendimento, pois envolve uma questão cultural e consequências distintas de um atendimento desenvolvido com ouvintes. Além do desconforto pelo receio de não serem entendidos, por erro no tratamento, e até perda da autonomia e privacidade quando na presença de um terceiro para viabilizar e facilitar a comunicação.

Igualar as pessoas no atendimento pode ser uma estratégia que parece não ser excludente ou preconceituosa, mas isso pode culminar no risco de evitar falar a respeito, do negacionismo, e acarretar em ainda mais invisibilidade para esse público, o que demonstra a necessidade de trabalhar com os profissionais, os conceitos e as práticas que envolvem a igualdade, a equidade, sua intercessão com a in/exclusão e o acolhimento.

À luz da Lei, os surdos gozam dos mesmos direitos e deveres dos ouvintes. Contudo, há políticas públicas que visam à equidade, para incluir surdos na sociedade e reparar uma exclusão histórica. Por isso, ao se pregar a igualdade entre surdos e ouvintes, excluem-se os surdos. A tentativa de minimizar o problema no discurso pode contribuir para a exclusão dessas pessoas.

Essa situação não se restringe a esse grupo, mas às pessoas com deficiência de um modo geral, quando se tenta diminuir os limites entre o que é normal e anormal, por interações sociais calculadamente desatentas com redução de contatos oculares que ampliam os afastamentos, aliviam o peso da observação do corpo do outro ^15^.

Esses fatos caracterizam representações, correspondendo a um recurso de diminuir a carga, o peso do problema, minimizá-lo. Não falar a respeito, silenciar o problema, colocando todos os envolvidos no mesmo bojo se apresenta como uma estratégia de lidar com a situação e finda por escamoteá-la. O discurso da igualdade, no caso, apresenta-se como uma armadilha, ao negar a diferença.

Outra reflexão pode ser feita pelo viés emocional. Tentar diminuir o problema à questão da comunicação pode amenizar o sentimento de culpa para o profissional por não atender da forma como gostaria. Os sentimentos de angústia, medo e impotência, acompanhados de irritabilidade e falta de paciência afloram. O profissional tem que lidar com todas essas emoções e ainda dar atenção e prestar cuidados aos usuários sem demonstrar seus sentimentos, para não gerar insegurança nem os deixar mais sensíveis do que já se encontram por precisarem de assistência à saúde. Os sentimentos negativos associados aos entraves na comunicação podem gerar prejuízo no aspecto emocional e afetivo, atingindo ambos os lados.

Verifica-se, ainda, a preocupação com o diagnóstico e o tratamento, ao expor a dificuldade de compreender o que o paciente sente, o que implica diretamente na sua ação diante do problema de saúde. Ademais, quanto mais distantes os profissionais se encontram do universo das pessoas surdas por eles cuidadas, maiores são os desconfortos quando lidam com essas pessoas ^16^.

É preciso reconhecer que o sofrimento atinge também os profissionais de saúde. Não reconhecer significa negligenciar as preocupações do profissional, além de desvalorizar seu esforço e dirigir a culpa das falhas no atendimento a eles ^17^. A falha existe, mas deve-se frisar que cabe ao Estado colocar em prática suas legislações, ou até mesmo revê-las para qualifica-las, caso seja necessário para aprimoramento da assistência.

Quando profissionais atendem surdos sem fornecer acessibilidade linguística promove-se integração, mas não inclusão ^18^. Integrar pressupõe que a pessoa tenha que se adaptar ao meio, insere a pessoa sem alterar a estrutura para recebê-la, ao passo que incluir envolve transformar o meio em respeito à diversidade, há uma mudança de perspectiva ^18^. No caso de pessoas surdas, quando há somente integração, elas que deverão ter a capacidade de entender o que foi expresso, e não o ambiente e a sociedade que se transformaram para transpor a barreira imposta para sua inclusão. Esse fato mostra carência de compreensão sobre o conceito de inclusão e sobre as práticas que a viabilizam, além da relevância da discussão desse tema na graduação e no cotidiano do trabalho em saúde.

Em relação ao conceito de acolhimento, acolher em sua aplicação no campo da saúde corresponde a reconhecer o que o outro apresenta como necessidade legítima e singular, objetiva a construção de relações de confiança, compromisso e vínculo entre as equipes/serviços, trabalhador/equipes e usuário com sua rede socioafetiva. Para realizá-lo, é necessária a escuta qualificada, considerando a avaliação de vulnerabilidade, gravidade e risco ^19^. Quando não ocorre da forma como é proposto, o acolhimento passa a ser fragmentado, podendo transformar-se em um instrumento de exclusão.

Não obstante, o acolhimento é mencionado pelos profissionais, genericamente, como oferta de atenção e consideração pelo usuário, sentido aproximado do que se entende no senso comum. Tanto que há dúvidas se o usuário surdo foi ou não verdadeiramente acolhido no atendimento, e até informação de impossibilidade de acolhê-lo em razão da barreira na comunicação.

O acolhimento e a inclusão se relacionam intimamente com a integralidade do cuidado, ao tempo que prevê superação da fragmentação deste e articulação das ações e serviços necessários em todos os níveis de complexidade ^20^. A relação entre esses conceitos na assistência contribui com a promoção de equidade, ao ofertar especificidades a públicos que as demandam, entendendo que a verdadeira igualdade somente será alcançada quando as diferenças forem reconhecidas e consideradas ao cuidar do outro. Acolher, incluir, atender às demandas proporciona cuidado integral, seja ele focalizado no encontro entre profissional/equipe/usuário ou ampliado, quando há boa conexão entre os serviços da rede ^21^.

Os resultados evidenciam que, nas representações dos profissionais há inclusão, por não negarem e realizarem o atendimento aos usuários surdos. O mesmo se observa em relação ao ato de acolher, que se associa ao esforço dos profissionais para realizar o cuidado, apesar do despreparo para lidar com usuários surdos. Todavia, os termos incluir e acolher na saúde ultrapassam a compreensão de seu cotidiano à luz do senso comum. No campo da saúde, acolher e incluir se respaldam em legislações e portarias, são conceitos teóricos e técnicos, amparados em princípios e diretrizes do Sistema Único de Saúde (SUS). O que se tem de fato é integração, mas não inclusão e com isso se constata a necessidade de reforçar o entendimento normativo desses termos na formação em saúde associado à prática, dialeticamente.

A dificuldade de acolher e incluir impacta negativamente no acesso de surdos aos serviços de saúde, em decorrência também da falta de acessibilidade em relação às informações de saúde, como medidas de prevenção de doenças e cuidados para promoção de saúde. A precariedade de informações em saúde para surdos, o sub diagnóstico e o sub tratamento podem colocá-los em riscos evitáveis e expectativa de vida reduzida ^22^.

As barreiras de comunicação afastam a pessoa surda das unidades de saúde, influenciam sua percepção sobre os atendimentos e a respeito da própria saúde ^23^. Destaca-se que, com a pandemia de COVID-19, os problemas vivenciados por surdos no serviço de saúde foram potencializados. A dificuldade de obter informações sobre a doença, acessar os serviços, ter acessibilidade para cuidado à saúde e de humanização, que antes já eram evidenciadas, expuseram o quanto estamos em dívida quando o assunto é a inclusão social de surdos e, principalmente, na saúde ^24^.

Por esses motivos é tão importante discutir sobre os conceitos de acolhimento, inclusão, integralidade, universalidade e equidade para a população em geral e também no contexto do cuidado a pessoas com deficiência, de forma que esses conceitos não fiquem apenas no campo das ideias, contemplando-os na prática, na assistência, nos espaços de educação permanente e em disciplinas na graduação, mesmo que de maneira transversal, conforme sugeridos pela Organização Mundial da Saúde (OMS), no *Relatório Mundial sobre a Deficiência* ^25^.

Os resultados também evidenciaram diferenças culturais no atendimento, como no caso do parto de mulheres surdas, que se debruça mais na experiência visual. A mãe surda vê se o filho está bem pela expressão facial do choro, enquanto com mães ouvintes o foco está no som do choro do bebê (intensidade e vigor). A compreensão de que há diferenças culturais também é reforçada quando os participantes estabelecem ancoragem da Libras com línguas estrangeiras, a exemplo do atendimento a haitianos.

A ancoragem possibilita que o acontecimento, ora desconhecido, seja acomodado no sistema de pensamento preexistente da pessoa que o interpreta ^26^. Tem o intuito de aproximar e tornar compreensível o que está fora da nossa capacidade de compreender de imediato. Assim, sem o domínio de um significado inteligível para o nosso sistema sociocognitivo, seria impossível lidar com a realidade posta, demandando recursos para dominá-la ^27^.

Sobre as estratégias utilizadas para estabelecer comunicação com pessoas surdas sinalizantes demonstra-se o quanto a dimensão prática das representações sociais se associa aos aspectos históricos e culturais que permeiam a surdez e as pessoas surdas. Quando a comunicação se concentra no Português escrito, na oralização/leitura labial, mímica e gestos, remete-se a um passado que ainda reflete no presente, desde 1880 com o Congresso de Milão, em que houve o fortalecimento da oralização e proibição do uso da língua de sinais. Estarmos tão aquém da inclusão linguística nos aponta o quanto ainda invisibilizamos as demandas dos surdos, o que também descortina o capacitismo.

Para realizar o atendimento, os profissionais se utilizam de um arsenal de possibilidades na tentativa de compreender as demandas expostas pelos usuários surdos, seja envolvendo terceiros para auxiliar no atendimento (acompanhantes, intérpretes), ou por meio de outros instrumentos, como Português escrito, leitura labial, aplicativos de tradução e gestos, ou seja, usam as estratégias que estão ao seu alcance no momento do atendimento.

Estudo anterior ^7^ mostrou que tais estratégias indicam ineficiência na comunicação para promover a participação plena de pessoas surdas sinalizantes, o que corrobora os achados desta pesquisa, ao ser apontado pela maioria dos profissionais a dificuldade de entendimento para realizar assistência, apesar do uso das estratégias supracitadas, sendo a experiência, inclusive, associada ao sentimento de frustração.

No que tange ao uso do Português escrito, ressalta-se que a língua de sinais se estrutura sintaticamente de forma distinta ao Português, o que por vezes pode dificultar tanto a compreensão do profissional de saúde ao ler algo redigido por pessoas surdas, quanto o contrário. O uso do Português escrito é a estratégia que mais dificulta a troca de informações no atendimento e esse fato pode constranger pessoas surdas sinalizantes, além do que a compreensão do Português escrito se relaciona com o nível de escolaridade desses sujeitos ^23^.

Em relação à leitura labial, em geral ela é superestimada pelos profissionais de saúde. Surdos que perderam a audição após o desenvolvimento da linguagem oral tendem a ter mais facilidade que surdos congênitos para compreensão ^5^. Ainda mais, não corresponde a uma estratégia tão segura em razão da existência de fonemas que possuem o mesmo ponto articulatório, o que pode resultar em falhas na comunicação. O uso de máscara também corresponde a um empecilho para a utilização dessa ferramenta.

A inclusão de acompanhantes no atendimento facilita a comunicação, mas apesar de favorecer a compreensão das necessidades de saúde, fere a privacidade, o sigilo, o protagonismo e a autonomia de usuários surdos no serviço de saúde, elementos fundamentais no processo de cuidar ^28^. Já no caso de mediação do atendimento por intérprete de língua de sinais, há apoio por parte dos surdos, porém com ressalvas, pelos mesmos motivos dos acompanhantes, somado à falta de confiança, escassez de profissionais, e até mesmo o desconhecimento por parte dos usuários sobre as obrigações legais para exigir a presença do profissional ^29^.

Embora exista a possibilidade de valorizar a comunicação não verbal, principalmente por ser visual, meio pelo qual os surdos sinalizantes apreendem o mundo, o uso de mímicas e gestos pode gerar falsa impressão de serem excelentes facilitadores da comunicação. Por serem pessoais e individuais pode haver falhas na comunicação, com entendimentos equivocados, por exemplo.

Atualmente, tem-se utilizado também o apoio de aplicativos para tradução da língua de sinais durante atendimentos a pessoas surdas, o que é útil no entendimento. O uso da tecnologia possibilita a melhoria da comunicação ^30^. Contudo, vale ressaltar que seu uso desconsidera elementos importantes na comunicação, como as expressões faciais. Ademais, é suscetível a falhas técnicas e pode levar ao descumprimento do disposto no *Decreto nº 5.626/2005* quanto à exigência de serviços disporem de profissionais aptos a utilizarem língua de sinais no atendimento.

As consequências também podem ser vistas na prática do cuidado, quando mesmo com essas estratégias o profissional não consegue se comunicar com excelência, o que interfere inclusive na qualidade do cuidado, devido à possibilidade de gerar problemas, tais como: incorrer em erros de tratamento e diagnóstico, ferir a dignidade do outro, causar sofrimento em ambos os envolvidos, tanto por não ser atendido da forma como deseja, com respeito às suas necessidades, quanto por não cumprir seu oficio respeitando os princípios do SUS, com humanização e acolhimento.

A comunicação mediada pela Libras é condição essencial para a aproximação com pessoas surdas sinalizantes, pois favorece a competência no cuidado e a satisfação do profissional, ao mesmo tempo que possibilita ao usuário interagir e ter suas demandas de saúde resolvidas. Ainda que a comunicação em Libras não seja fluente, o fato de ter suas necessidades compreendidas possibilita uma comunicação interpessoal mais afetiva, logo, efetiva e empática. Por conseguinte, a formação em saúde perpassada pelo aprendizado de Libras e pela convivência com pessoas surdas sinalizantes, sendo uma potência na formação, ao viabilizar o aperfeiçoamento de capacidade crítica, reflexiva e de compromisso social ^30^.

Percebe-se que a formação em Libras, seja por curso livre ou disciplina de Libras, não tem sido suficiente para instrumentalizar profissionais de saúde para o atendimento a surdos usuários de língua de sinais. Mesmo os participantes que realizaram formação básica em Libras apresentam dificuldades no atendimento e utilizam recursos insatisfatórios para a inclusão de pessoas surdas. Esse fato corrobora os resultados de um estudo ^31^ que verificou fragilidades na formação dos profissionais em relação à disciplina de Libras, evidenciadas pela falta de padronização quanto aos períodos ofertados e à reduzida carga horária.

Por outro lado, atitude diferenciada, inclusivista, foi majoritária nos resultados dessa pesquisa, oriundos dos participantes que informaram ter convívio com pessoas surdas e formação específica. Logo, evidencia-se que o elemento de maior impacto para o cuidado à saúde de pessoas surdas sinalizantes é oriundo dessas duas condições.

Há, portanto, falha do Estado em relação ao acesso e à acessibilidade da pessoa surda no serviço de saúde e na formação dos profissionais nessa área. Porém, essa realidade tende a mudar, graças às lutas dos movimentos surdos e conquistas advindas das políticas públicas inclusivistas. Pessoas surdas têm tido mais acesso à educação, a níveis escolares mais altos, como graduação e pós-graduação. O conhecimento, as relações sociais e as experiências empoderam as pessoas e por isso, também, tem ocorrido judicializações que tratam de inclusão na saúde, além de maior engajamento a respeito de inclusão de surdos nas redes sociais e nos programas de televisão.

As representações sociais guardam relações entre a ciência e a vivência, remetendo para a pesquisa das relações entre o pensamento natural ou do senso comum e o pensamento científico, da difusão dos conhecimentos e a transformação de um tipo de saber em outro ^8^ ^,^ ^26^. É essa relação dialética entre o que se apresenta como necessidade advinda da prática assistencial, dos conhecimentos prévios compartilhados no senso comum, das informações que circulam na mídia e redes sociais que formam as representações sociais, e essas representações permitem reflexões que, quando acionadas e entendidas como oportunidade, podem servir de diagnóstico e de ponto de partida para transformações necessárias para o coletivo.

## Considerações finais

Por meio deste estudo foram analisadas as representações do cuidado a pessoas surdas usuárias de língua de sinais por profissionais de saúde associadas, sobretudo, em sua dimensão simbólica, à interdição da relação pela falta de entendimento entre as partes durante o processo de cuidar; da dimensão afetiva, marcada por sentimentos de frustração e dificuldade; dimensão comportamental e dimensão atitudinal, ao recorrer a acompanhante, intérprete, português escrito, aplicativos de tradução/internet e gestos.

Apesar de sensibilizar o profissional de saúde sobre as necessidades e singularidades desse grupo, a formação em Libras, por curso livre ou disciplina, não tem sido suficiente para instrumentalizar profissionais de saúde para o atendimento à pessoa surda. A atitude diferenciada, inclusivista, adveio de profissionais que informaram ter convívio com pessoas surdas, associado à formação específica. Portanto, evidencia-se que o elemento de maior impacto para o cuidado à saúde da pessoa surda sinalizante é oriundo dessas duas condições.

Como limitações do estudo, tem-se a amostra reduzida focalizada em uma unidade de saúde. Ampliar a pesquisa em localidades com instituições de ensino superior com disciplina de Libras na graduação em saúde com carga horária maior que 80h e com campo prático de atendimento a surdos pode ajudar a compreender melhor seu impacto na prática assistencial dos profissionais de saúde.
